# Supplementary material for: Differences in Cytokine Expression at Baseline and in Response to Mineral Stimulation by Peripheral Blood Mononuclear Cells from Podoconiosis Cases and Healthy Control Individuals
Source: Trop Med Infect Dis. 2024 Oct 22;9(11):252. doi: 10.3390/tropicalmed9110252 (PMC11598685; doi:10.3390/tropicalmed9110252)
Supplement: Supplementary file 1 [file tropicalmed-09-00252-s001.zip › tropicalmed-3221516-supplementary.pdf]

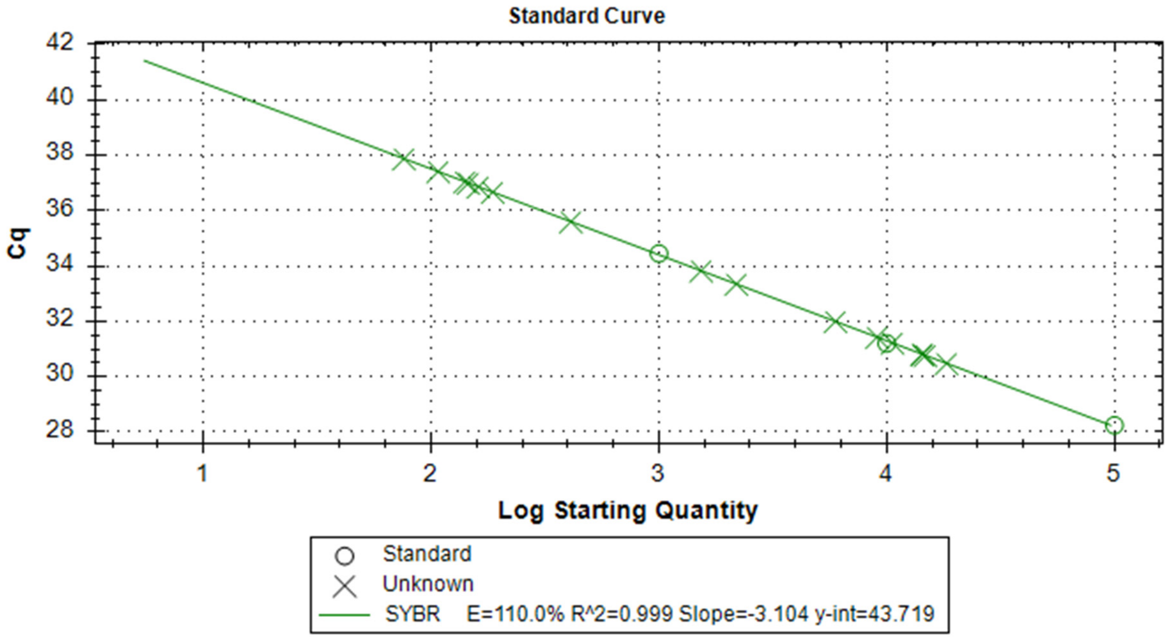

Supplementary figure S1. Standard curve graph showing the efficiency and correlation of the qRT-PCR experiment.

A ten-fold serial dilution of one sample was performed starting from stock concentration, to 1:10, 1:100. 1:1000, and 1:10.000, in order to extrapolate the efficiency, the correlation and the slope of the assay. The y axis shows the Ct value while the x axis shows log starting quantity for the standard and the unknown sample.
